# The Role of Biofilm-Derived Compounds in Microbial and Protozoan Interactions

**DOI:** 10.3390/microorganisms14010064

**Published:** 2025-12-27

**Authors:** Smruti Mahapatra, Serge Ankri

**Affiliations:** Department of Molecular Microbiology, Ruth and Bruce Rappaport Faculty of Medicine, Technion, Haifa 31096, Israel; smruti.m@campus.technion.ac.il

**Keywords:** biofilm, protozoa, interspecies interactions, metabolites, host–pathogen relationships

## Abstract

Biofilms are more than just structural microbial communities. They are dynamic chemical ecosystems that synthesize a range of extracellular compounds involved in functions that extend beyond biofilm architecture. From quorum-sensing molecules like acyl-homoserine lactones (AHLs) to short-chain fatty acids (SCFAs), phenazines, indoles, and reactive sulfur species (RSS), biofilm-derived metabolites can impact the physiology and behavior of microorganisms living in the same ecosystem, including other bacteria and protozoa. It has recently been demonstrated that such molecules may also modulate competition between microbes, promote cooperation, and impact motility, differentiation, or virulence of free-living and parasitic protozoa. This review aims to discuss biofilm compounds that mediate interspecies or interkingdom interactions and their involvement in regulating gut and environmental microbiomes functions, and host–pathogen relationships with special emphasis on protozoan activity and the infection outcome. This review will also address how this chemical dialog can be explored to identify new therapeutic interventions against microbial infections and parasitic diseases.

## 1. Introduction

Biofilms are highly organized microbial communities embedded in a self-produced extracellular matrix (ECM) consisting of polysaccharides, proteins, lipids, and extracellular DNA (eDNA) [1]. Microorganisms mostly form biofilms in their natural and host-associated habitats rather than being in their planktonic form [2]. Biofilms have been viewed mostly in relation to surface attachment [3] and antimicrobial resistance [4]; however, they are now increasingly understood as biochemical reactors which influence microbial activity through secretion of diverse bioactive compounds [5].

While the canonical view emphasizes the role of secreted molecules in maintaining biofilm architecture and community integration [6], evidence shows that these metabolites extend their influence beyond the biofilm, mediating cross-species and even cross-kingdom interactions [7]. Compounds such as acyl-homoserine lactone (AHL), autoinducer-2 (AI-2), phenazines, indole and short-chain fatty acids (SCFAs) can diffuse into the environment and modify gene expressions, stress responses, motility and various metabolic pathways in surrounding microbes [8,9,10,11,12]. Such effects can be either cooperative or competitive, contributing to community structuring, exclusion of rivals, or facilitation of syntrophic interactions of microbes.

In addition to intra- and intermicrobial interactions, bacteria in biofilms must also defend themselves from natural predators, particularly protozoan grazers. Unlike planktonic cells, biofilm-associated bacteria lack the ability to escape predators, making them especially vulnerable to grazing [13]. A recent study by Kolodkin-Gal et al., showed that planktonic cells exposed to the ‘*Entamoeba histolytica*’ lysate deactivate the expression of genes associated with biofilm formation while inducing their motility to avoid predation [14]. At the community level, mixed-species biofilms often display greater tolerance to protozoan grazing than single-species biofilms. For predation-sensitive bacterial species, integration into a mixed-species biofilm is an advantageous strategy, as they can be shielded by predation-resistant partners and by the shared protective environment of the consortium [15]. In response to the dual ecological pressures of competition and predation, many bacteria have evolved to secrete metabolites that serve both defensive and offensive functions. Thus, the secretion of these bioactive molecules represents a multifaceted survival strategy, enhancing microbial fitness by mediating both interspecies competition and defense against eukaryotic grazers.

Given these interactions, microscopic eukaryotes, mainly protozoa, remained underappreciated performers in such dialogs. Free-living protozoa such as Acanthamoeba and Dictyostelium engage in biofilm grazing, chemotaxis, and avoidance behaviors mediated by bacterial products, while some biofilms actively repel these predators through toxic or repellent compounds or by altering their matrix architecture to reduce accessibility [16,17,18,19]. Likewise, parasitic protozoa such as *E. histolytica* and *Giardia lamblia*, which inhabit host-associated microbiomes, are also influenced by biofilm-derived molecules. Recent findings indicate that SCFAs can modulate differentiation processes in these parasites [20,21,22]. The focus of this review is on the relatively new idea of biofilms as dynamic chemical communication networks whose metabolites interact with both neighboring bacteria and protozoa. We intend to categorize the main classes of biofilm compounds, outline their physiological actions, and assess their effects, if any, on competitors among the bacteria and responders from the protozoa. We also analyze the ecological and biomedical relevance of these interactions in the gut microbiota, environmental microbiomes, and polymicrobial infections. By considering biofilms to be the main factors of intermicrobial communications, we hope to provide new insights toward the control of sophisticated microbial consortia.

## 2. Survey Methodology

Literature research is aimed at collecting any published data about biofilm-secreted compounds, their effects on bacterial competitors, and impact on protozoa. We searched for literature relevant to the topic of articles using PubMed and Google Scholar. The included keywords were as follows: biofilms, quorum sensing, interspecies interactions, metabolites, host–pathogen relationships, protozoa, Acanthamoeba, and *E. histolytica*. We limited our selection to peer-reviewed articles, including primary research and reviews that reported experimental data. Screened articles were used as references for this review.

## 3. Biofilm-Secreted Compounds: Classification and Mechanisms

Based on their chemical nature and functional roles we classify these compounds into six major categories: quorum sensing (QS) molecules, secondary metabolites, antimicrobial peptides, exopolysaccharides and eDNA, redox-active molecules, and outer membrane vesicles (OMVs).

### 3.1. QS Molecules

QS is a sophisticated communication mechanism that enables bacteria within biofilms to coordinate gene expression based on population density, thereby enhancing collective survival strategies [23]. This process primarily depends on the production and detection of small signaling molecules known as autoinducers (AIs). As these molecules accumulate and reach a threshold concentration, they trigger transcriptional responses that regulate key behaviors such as biofilm formation, motility, virulence, and antibiotic resistance. In Gram-negative bacteria, acyl-homoserine lactones (AHLs) serve as the main QS signals, whereas Gram-positive bacteria typically employ autoinducing peptides (AIPs) [24]. AI-2, synthesized via the LuxS pathway, functions as a broadly conserved signal recognized across bacterial species [25]. Beyond their canonical roles in bacterial communication, QS signals can influence other organisms. For instance, AHLs have been shown to suppress motility and virulence factor expression in *Escherichia coli* [26,27], and AI-2 can modulate host immunity and shape gut microbiota composition [28]. Interestingly, eukaryotic microbes such as *Acanthamoeba castellanii* exhibit chemotactic responses to bacterial products, as formyl-methionyl-leucyl-phenylalanine (fMLP), lipopolysaccharide, and lipid A were statistically significant, as was the response to fMLP benzylamide, suggesting that QS signals may mediate interkingdom interactions [29].

### 3.2. Secondary Metabolites

Secondary metabolites such as phenazines, indole, hydrogen cyanide, and volatile organic compounds (VOCs) are often secreted by biofilms and can serve antimicrobial, signaling, or redox-regulatory functions [30,31]. Phenazines produced by *Pseudomonas aeruginosa*, including pyocyanin, act as redox-active compounds that modulate iron acquisition and suppress competitors [32]. Indole, secreted by *E. coli* and other gut microbes, has broad effects on stress responses, antibiotic resistance, and biofilm formation function in bacteria [33], and regulation of motility and virulence in protozoa such as *E. histolytica* [34].

### 3.3. Antimicrobial Peptides and Bacteriocins

Bacteriocins and antimicrobial peptides (AMPs) secreted within biofilms play roles in competitive exclusion by targeting closely related strains or species. For example, Streptococcus mutans secretes mutacins that inhibit competing oral streptococci [35]. In mixed-species biofilms, bacteriocin production can modulate community composition and resist protozoan predation by excluding vulnerable strains [36,37].

While their effects on protozoa are less well studied, some bacteriocins can directly target the protozoan predators. For example, *Janthinobacterium lividum* and *Chromobacterium violaceum* release the bioactive metabolite violacein upon digestion and cause rapid death and lysis of flagellate predators [37].

### 3.4. Exopolysaccharides (EPS) and eDNA

EPS are fundamental components of the biofilm matrix, playing essential roles in biofilm formation, structural integrity, and defense against environmental stresses. Composed mainly of complex sugars, EPS promotes microbial adhesion to surfaces, intercellular cohesion, and protection from antimicrobial agents and host immune responses. For example, *P. aeruginosa* produces three biofilm exopolysaccharides-alginate, Pel and Psl, and among these alginate enhance biofilm resistance in cystic fibrosis lung infections [38,39]. Similarly, in *Staphylococcus epidermidis*, polysaccharide intercellular adhesin (PIA) is crucial for biofilm accumulation on medical devices [40]. In *Bacillus subtilis*, the protein TasA works alongside EPS to form robust biofilms, with TasA forming amyloid-like fibers that stabilize the matrix and support multicellular organization [41,42,43]. Interestingly, protozoan grazers such as *E. histolytica* may recognize and interact with these EPS components during biofilm predation. For instance, research by Ankri and colleagues demonstrated that *E. histolytica* cysteine protease (CPs) enzymes are capable of cleaving TasA, suggesting a targeted mechanism to disrupt biofilm structure and facilitate grazing [44]. This raises the possibility that protozoa detect specific EPS molecules or associated proteins as cues to locate and degrade biofilms, making this an intriguing area for further exploration into predator–prey dynamics within microbial communities.

eDNA has emerged as a key factor in biofilm development, acting not only as a structural scaffold but also influencing bacterial behavior. It facilitates initial attachment and biofilm initiation in pathogens such as *Listeria monocytogenes*, *Campylobacter jejuni*, *Helicobacter pylori*, and *Staphylococcus aureus*, where it acts as an electrostatic net that tethers cells together. In *P. aeruginosa*, eDNA acidifies the local environment and triggers resistance mechanisms via PhoPQ and PmrAB regulatory systems, enhancing protection against aminoglycosides and antimicrobial peptides [45]. Beyond structural roles, eDNA can function as both a chemoattractant, recruiting new cells [46], and a chemorepellent, as observed in *Caulobacter crescentus*, where it deters newborn cells from joining mature biofilms and promotes dispersal. Although there is no direct evidence that protozoa detect eDNA, it is conceivable that eDNA influences their grazing behavior. Protozoa might preferentially avoid eDNA-rich regions due to increased biofilm cohesion or altered pH, or conversely, be attracted to areas where eDNA signals the presence of active microbial communities [47].

### 3.5. Redox-Active Molecules and Reactive Oxygen Species (ROSs)

Biofilms can generate ROSs either as metabolic byproducts or through specialized mechanisms involving redox-active compounds [48]. Phenazines and flavins function as electron shuttles within biofilms, modulating the redox state of neighboring microbes and potentially inducing oxidative stress or inhibiting growth [49]. These redox-active metabolites can suppress sensitive bacterial competitors in polymicrobial communities [50,51]. The toxicity of phenazines such as 1-hydroxyphenazine, phenazine-1-carboxylic acid, and pyocyanin to nematodes [52], and of 5-methyl-phenazine-1-carboxylic acid to fungi [53], suggests that these compounds may also play a role in controlling protozoan predators.

While flavins are known to be essential cofactors for many protozoan enzymes, such as thioredoxin reductase and flavin reductase in Giardia, where inhibition of these enzymes confers resistance to metronidazole [54], or in *E. histolytica*, where downregulation of thioredoxin reductase activity (which requires FAD as a cofactor) increases susceptibility to metronidazole [55], their direct toxicity to protozoa remains poorly investigated. An exception is their use as photosensitizers; for instance, in the treatment of dermal leishmaniasis, riboflavin and FMN, when activated by blue light, have been shown to kill Leishmania promastigotes and amastigotes by inducing OS [56].

### 3.6. OMVs

OMVs are spherical lipid structures secreted by Gram-negative bacteria, often enriched in toxins, signaling molecules, DNA, and enzymes. Biofilm-derived OMVs can influence quorum sensing, modulate host responses, and promote antibiotic resistance [57,58,59,60]. OMV uptake by host cells involves multiple factors, including surface molecules like LPS and the O-antigen. OMVs without O-antigen enter via clathrin-mediated endocytosis, while those with it use raft-dependent pathways. PAMPs on OMVs can trigger TLR signaling, promoting internalization and LPS delivery. Some OMVs, such as those from *Legionella pneumophila* or *C. jejuni*, can fuse with host membranes [61,62]. Overall, uptake is influenced by OMV composition, size, and environmental conditions. Although the interactions between bacterial OMVs and protozoan predators grazing on biofilms remain largely unexplored, the known ability of OMVs to interact with various eukaryotic cells, together with the important roles that extracellular vesicles from unicellular parasites play in parasite–parasite communication and parasite–host interactions [63,64], suggests that bacterial OMVs may similarly influence the physiology and behavior of protozoa feeding on biofilms, potentially modulating their grazing activity or cellular functions.

## 4. Effects of Compounds Secreted by Biofilms on Bacterial Competitors and Protozoan Predators

Building on the classification outlined above, this section discusses how biofilm-secreted compounds influence microbial cooperation and competition, and how these molecules affect protozoan predators by altering their physiology and survival.

### 4.1. SCFAs

SCFAs are key microbial metabolites composed of one to six carbon atoms, primarily generated through the fermentation of dietary fibers by gut bacteria [65]. Among these, acetate, propionate, and butyrate are the most abundant, typically present in a molar ratio of 60:20:20 [66]. Acetate is produced by anaerobic bacteria such as *Akkermansia muciniphila* and Bacteroides spp., while propionate is synthesized via the succinate pathway. Butyrate, largely produced by commensal bacteria like *Faecalibacterium prausnitzii* and Clostridium clusters IV and XIVa, not only supports microbial community structure but also serves as a primary energy source for intestinal epithelial cells [65].

SCFAs strongly influence microbial competition, especially under nutrient limitation. In co-culture, *Bacteroides thetaiotaomicron* and Roseburia intestinalis both ferment glucose and pyruvate to produce acetate, lactate, and formate. However, *B. thetaiotaomicron* also generates succinate, which acidifies the medium and causes its own decline, while R. intestinalis switches to metabolizing lactate and acetate after glucose depletion, sustaining growth and eventually outcompeting *B. thetaiotaomicron*. Thus, SCFAs can act as toxic byproducts for some species but valuable energy sources for others, depending on their metabolic adaptability of the producing species [67].

Protozoan colonization can influence the abundance of SCFA-producing bacteria. For instance, colonization with the commensal protozoan Blastocystis ST4 enriches SCFA producers such as Ruminococcaceae and Roseburia, contributing to a more balanced gut microbiota. Whereas pathogenic protozoa like *Cryptosporidium parvum* are often associated with reduced SCFA levels in the gut [68]. SCFAs themselves directly impact protozoan development. For example, acetate and propionate promote *E. histolytica* encystation but inhibit this process in *Entamoeba invadens*, highlighting species-specific responses [20,21]. Similarly, butyrate inhibits *C. parvum* sporozoites in vitro, and derivatives such as valproate and 4-phenylbutyrate suppress *Toxoplasma gondii* proliferation and reduce brain cyst burden in vivo [69,70]. However, the specific function of SCFs within biofilm environments remain largely unexplored, as most studies have focused on host or planktonic systems rather than multicellular microbial consortia.

### 4.2. AHLs

Among the diverse biofilm-derived molecules, AHLs plays a central role in QS systems, enabling bacteria to coordinate group behaviors such as biofilm formation, antimicrobial production, stress responses, and influence bacterial competition and cooperation. For example, in *Burkholderia thailandensis* and *C. violaceum* co-culture, both species grow independently, but as the culture matures, QS-regulated antimicrobials are secreted, resulting in a dramatic collapse of *C. violaceum* populations and a partial decline in *B. thailandensis*. Mutants lacking AHL synthesis lose their competitiveness, but supplementation with the appropriate AHLs restores this balance, demonstrating the essential role of QS during competition. Interestingly, *C. violaceum* can “eavesdrop” on AHLs produced by *B. thailandensis*, particularly C8-HSL, using its own CviR receptor. This cross-species QS allows it to activate violacein production and enhance its survival [71]. Moreover, AHLs may influence interspecies interactions in more indirect ways. Long-chain AHLs produced by *P. aeruginosa* can interfere with the agr QS system of *S. aureus*, resulting in increased expression of surface adhesins such as FnbAB and ClfB. This modulation enhances *S. aureus* biofilm formation and host invasion, suggesting that AHLs can reshape the behavior of even non-AHL-producing bacteria, thereby altering community structure and possibly their susceptibility to protozoan predation [72,73,74].

Beyond regulating bacterial competition, AHL-mediated QS becomes evident in predator–prey interactions within biofilms. In *P. aeruginosa*, the Las and Rhl systems regulate the expression of several toxic compounds, such as elastases, hydrogen cyanide, pyocyanin, exotoxin A, and rhamnolipids, with these compounds collectively forming a potent defense system [75]. QS-deficient mutants lacking the receptors LasR or RhlR show reduced toxicity toward protozoan grazers, especially during biofilm maturation. Among these compounds, RhlA-derived rhamnolipids have been demonstrated to deter predators such as *Dictyostelium discoideum* and *Tetrahymena pyriformis* [76,77]. Interestingly, this defensive role of AHLs can vary across species and biofilm developmental stages. In *Serratia marcescens*, QS was found to be dispensable during early biofilm formation but critical during later stages under flow conditions, where AHL signaling (particularly through BHL) directed the development of filamentous biofilms that were resistant to grazing [78]. This information highlights how QS-regulated defenses are modulated depending on the ecological context and physical structure of the biofilm, enabling bacterial communities to resist protozoan predation.

### 4.3. Phenazines

Within the stratified architecture of a biofilm, oxygen is rapidly depleted by surface-associated cells, creating a gradient that makes the deeper regions hypoxic, while oxidant limitation promotes colony wrinkling, which increases the access to oxygen in the atmosphere for resident cells [79]. To overcome this limitation, bacteria produce phenazines that balance the intracellular redox state under low-oxygen conditions. This redox buffering also influences colony morphology specifically by inhibiting wrinkling, and this response otherwise enhances oxygen diffusion into the colony [80].

Beyond internal redox homeostasis, phenazines serve as potent chemical weapons in microbial competition. In polymicrobial biofilms, *P. aeruginosa* produces phenazines such as pyocyanin, which suppress competitors like *S. aureus*. Pyocyanin disrupts membrane integrity and generates reactive oxygen species (ROSs), overwhelming the antioxidant defense systems of *S. aureus* in both planktonic and biofilm-associated states [81,82,83,84,85,86]. Similarly, *Pseudomonas chlororaphis* YL-1 produces phenazine-1-carboxylic acid (PCA), which inhibits the growth of *Acidovorax citrulli* by increasing intracellular ROS levels and catalase activity, contributing to oxidative stress in the target cells [87].

Phenazines also exert strong antiprotozoal effects, contributing to biofilm defense against grazers. Recent work by Ghergab et al. [88] showed that phenazines produced by *P. chlororaphis* PA23 directly kill *A. castellanii* as a stress response. and upregulate quorum-sensing genes (phzI and phzR) along with the antimicrobial metabolite pyrrolnitrin. These findings indicate that the presence of protozoan predation can trigger a coordinated chemical defense response in phenazine-producing bacteria [88,89]. Thus, phenazine has dual functionality within biofilms, serving as metabolic adaptors in oxygen-limited environments and as offensive agents against both bacterial competitors and protozoan predators.

### 4.4. Indole

Originally considered a simple byproduct of tryptophan catabolism, indole has since emerged as a critical interkingdom signaling molecule that shapes bacterial physiology and community behavior. It is synthesized by the enzyme tryptophanase (TnaA), whose expression increases under nutrient limitation and stationery-phase conditions [90,91].

In structured microbial communities, indole plays a multifaceted role in bacterial competition and cooperation. In co-culture systems, *E. coli* mutants unable to synthesize indole show a reduced competitive fitness against *P. aeruginosa*, and this characteristic is reversed upon indole supplementation [92]. However, the effects of indole are not universally beneficial; when Pseudomonas strains engineered to express toluene o-monooxygenase (that degrades indole to an insoluble indigoid) were co-cultured with *E. coli*, a higher number of *E. coli* cells were observed in the biofilm compared to co-culture with wild-type Pseudomonas [93]. This suggests that under certain conditions, indole may act as a stress-inducing molecule, limiting the growth of some species, and that enzymatic degradation of indole may relieve this inhibition.

Indole also contributes to biofilm defense against protozoan predators. In Vibrio cholerae, deletion of the tnaA gene, which encodes the enzyme responsible for indole production, leads to increased susceptibility to grazing by *D. discoideum*. Supplementation with exogenous indole partially restores resistance, but this effect depends on an intact vas operon, suggesting that indole triggers downstream defense mechanisms rather than acting as a direct toxin [94]. Consistent with this, higher fecal indole levels correlate with reduced *C. parvum* burden in humans [11,95]. Conversely, recent work by Zanditenas et al. [34] demonstrated that while indole is initially toxic to *E. histolytica* trophozoites, prolonged exposure to indole enables *E. histolytica* to adapt, becoming more motile, virulent, and resistant to oxidative stress. This highlight indole’s dual nature, initially acting as an antiparasitic cue but ultimately produce more resilient protozoan populations.

### 4.5. Violacein

Violacein is a purple bisindole alkaloid, produced by bacteria such as *C. violaceum*, *J. lividum*, *Pseudoalteromonas luteoviolacea*, and *Alteromonas luteoviolacea* [96,97,98,99]. It is synthesized from two L-tryptophan molecules through the vioABCDE operon [100]. Violacein production commonly accompanies biofilm formation and is concentrated in biofilm-associated cells, where it contributes to microbial defense and interspecies interactions [101].

Violacein has broad-spectrum antibacterial properties, contributing to microbial competition within biofilms. It has bacteriostatic activity against both Gram-positive and Gram-negative bacteria, including *S. aureus* ATCC 29213, MRSA (ATCC 43300) [102], and *Clavibacter michiganensis*, likely by disruption of membrane-associated ATPase activity [103]. Violacein from *J. lividum* supress the growth of *E. coli*, *P. aeruginosa*, *S. aureus*, *MRSA*, *Micrococcus luteus*, and *B. subtilis* [104]. In *C. violaceum*, violacein is primarily secreted via outer membrane vesicles (OMVs), which inhibit *S. aureus* in co-culture, whereas OMVs from a vioABCDE knockout strain lack this effect [105]. Interestingly, the same OMVs did not inhibit *E. coli*, contrasting with *J. lividum*-derived violacein, exhibit activity against *E. coli*. This highlights possible species-specific differences in violacein transport, release, and microbial targeting mechanisms.

Violacein also exhibits strong antiparasitic and antipredator activities. It inhibits *Leishmania amazonensis* in a dose-dependent manner and suppresses both chloroquine-sensitive and resistant *Plasmodium falciparum* strains, reducing parasitemia and improving survival in infected mice [106,107]. Violacein from *C. violaceum* and *J. lividum* rapidly kills flagellates such as *Ochromonas* sp. and *Spumella* spp. after ingestion of only 2–3 bacterial cells, causing cessation of flagellar beating, swelling, and lysis within hours [37]. Biofilms of *Pseudoalteromonas tunicata* producing violacein also eliminate the flagellate *Rhynchomonas nasuta*, whereas vioA mutants are readily grazed. This toxicity extends to amoebae (*A. castellanii*, *Acanthamoeba polyphaga*), ciliates (Tetrahymena, Euplotes), and other flagellates (*Cafeteria roenbergensis*), with nanomolar concentrations sufficient to induce a conserved programmed cell death-like response [101].

### 4.6. Reactive Sulfur Species (RSSs)

RSSs including hydrogen sulfide (H_2_S) and cysteine persulfide (Cys-SSH), are reactive metabolites involved in various biological and chemical processes [108]. They are produced across many microbial habitats; tongue biofilms, which generate volatile H_2_S, contributing to malodor and sewer biofilms induced concrete corrosion [109,110].

RSSs also modulate bacterial interactions; for example, *Salmonella Typhimurium* produces H_2_S via its phsABC operon, and although it does not enhance the bacterium’s own antibiotic resistance, the released H_2_S sensitizes *Enterococcus faecalis* and *Enterococcu faecium* to antibiotics [111]. Thus, even toxic sulfur species can act as ecological weapons, reshaping competition.

Beyond bacterial competition, RSSs also exhibit antiprotozoal activity. Exposure of *E. histolytica* trophozoites to either H_2_S or Cys-SSH induces rapid cytotoxic effects. H_2_S also inhibits *P. falciparum* growth and metabolism in vitro [112,113]. These effects highlight sulfur-based metabolites as conserved chemical defenses that regulate both microbial competition and protozoan survival.

## 5. Protozoan Responses to Biofilm-Derived Compounds

Biofilm-derived compounds can modulate protozoan physiology, inhibit motility, impair encystation, and induce cytotoxic effects. By acting through mechanisms such as oxidative stress induction, post-translational modifications, or disruption of mitochondrial function, these metabolites enable bacteria to inhibit digestion by predators and persist within competitive polymicrobial environments. This section summarizes the current understanding of how major classes of biofilm-secreted metabolites affect protozoan predators and influence interkingdom dynamics.

### 5.1. SCFAs

Within the host colon, Entamoeba trophozoites encounter high concentrations of SCFAs that act as environmental cues modulating their life cycle. Butyrate enters Entamoeba trophozoites via passive diffusion, which is pH-dependent, and its intracellular levels likely fluctuate along intestinal pH gradients [114]. Exposure to butyrate induces histone hypoacetylation, suggesting that Entamoeba histone-modifying enzymes are unusually sensitive to SCFAs. However, transcriptomic analyses reveal minimal gene expression changes after SCFA treatment, raising the possibility that SCFAs may not exert a major influence on gene expression at the transcriptional level in vivo or that axenically cultured parasites have lost the ability to respond robustly to these host-derived signals [114,115]. Although SCFAs inhibit encystation, the underlying mechanism remains unresolved and may depend on non-transcriptional regulation controlling the transition from trophozoite to cyst [115,116,117,118].

### 5.2. AHLs

Since eukaryotic and prokaryotic cells have coevolved, molecular signals from one domain can influence the other. In this context, bacterial QS molecules have been shown to affect eukaryotic cells by interkingdom signaling [119]. Most studies investigating the effects of AHLs on eukaryotic systems have focused on mammalian cells, particularly on the well-characterized 3-oxo-C12-HSL, C4-HSL from *P. aeruginosa*, and 3-oxo-C6-HSL from *Vibrio fischeri*. The structural feature enables smaller AHLs to freely diffuse across eukaryotic cell membranes, while longer-chain AHLs may require active transport mechanisms [120].

In mammalian systems, AHLs have been shown to enter cells and localize to both the nucleus and cytoplasm, depending on the cell line [121]. Furthermore, 3-oxo-C12-HSL can modulate signal transduction pathways and alter immune responses in both in vitro and in vivo settings [122]. For example, OdDHL from *P. aeruginosa* has been reported to inhibit proliferation and induce apoptosis in human breast cancer cells [123].

Evidence suggests that AHL-producing bacteria may affect protozoan behavior. In the stationary phase, when nutrient depletion slows bacterial growth and predation becomes a greater threat, wild-type QS-proficient strains exhibit increased resistance to protozoan grazing, compared to their quorum-sensing mutants [75]. Despite these behavioral observations, the molecular mechanisms by which protozoa detect or respond to AHLs remain elusive. This represents a significant gap in our understanding of interkingdom communication and protozoan-microbial interactions.

### 5.3. Phenazine

Phenazine production provides the producer with a competitive advantage by protecting them from predators and competitors. These compounds, produced mainly by metabolically slow or stationary-phase cells, contribute to long-term environmental persistence [124,125,126,127].

While the exact mechanisms through which phenazines affect protozoan cells remain poorly understood, several lines of evidence suggest that they exert toxic effects due to its structure and mode of action. Phenazines have a planar structure, and they are hydrophobic in nature, and their derivatives are likely capable of penetrating cellular membranes and may intercalate into DNA [127]. Phenazines, such as Myxin [128], iodinin [129], and pyocyanin [130], have been shown to inhibit DNA template-directed RNA synthesis either by direct DNA intercalation, interference with RNA polymerase, or binding to ribonucleoside 5′-triphosphates. The mode of action of phenazines depends on their ability to engage in redox cycling, and they can donate or accept electrons depending on the surrounding redox potential. This redox activity leads to the formation of ROSs, which can induce oxidative stress and cellular damage [131]. In protozoa, such oxidative stress may contribute not only to cell death but also to stress-induced responses such as encystation. However, whether phenazines directly enter protozoan cells or act primarily through extracellular interactions remains to be fully elucidated.

### 5.4. Indole

Indole is known for its role in regulating the transition from exponential to stationary phase during bacterial growth. Indole synthesized by producer bacteria is exported into the extracellular environment, where its accumulation can be sensed by nearby cells. Due to its small size and hydrophobic nature, indole and many of its derivatives can freely diffuse across lipid membranes, allowing passive uptake by sensitive cells [132].

One well-characterized example of indole’s antiparasitic activity is its effect on *C. parvum*. *C. parvum* lacks a conventional mitochondrion and possesses an organelle called the mitosome, which is not involved in energy production. *C. parvum* is also deficient in key metabolic pathways such as the TCA cycle and oxidative phosphorylation and is therefore heavily dependent on host-derived ATP to support its energy needs [133,134]. Indole can disrupt physiology of *C. parvum* by reducing the membrane potential of the mitosome. Additionally, indole exposure delays the life cycle progression of *C. parvum* in vitro and reduces infection severity in mouse models [135]. The antiparasitic effect of indole is not limited to direct action on the parasite; it also inhibits host cell mitochondrial respiration, leading to decreased intracellular ATP levels. This reduction in host ATP availability further limits *C. parvum* survival, since the parasite is dependent on host ATP for growth and replication [135].

### 5.5. Violacein

The precise mechanism by which violacein interacts with and kills protozoan cells remains largely unexplored. One of the main challenges in studying violacein’s cellular effects is its poor solubility in aqueous environments. To overcome this limitation, some bacteria pack violacein into OMVs, which enhance its stability and delivery [105,136]. This mode of transfer has been confirmed by Kowalska et al., who demonstrated that *J. lividum* EVs can interact with mammalian skin cells and deliver violacein [137].

Although direct evidence in protozoa is lacking, studies in *P. falciparum* provide insights on the mode of actions of violacein. Violacein binds to parasite’s major chaperons PfHsp90 and PfHsp70-1 [138], inhibiting their ATPase activity and disrupting protein folding, which leads to proteasomal degradation and disrupts proteostasis [139]. Additional mechanisms of violacein toxicity have been described in bacterial and mammalian systems; in *B. subtilis* and *S. aureus*, violacein causes severe membrane disruption, producing visible holes and promoting leakage of intracellular ATP. These effects point to direct damage to the cytoplasmic membrane as a bactericidal mechanism [140]. In mammalian cells, violacein exhibits cytotoxicity through several pathways. It increases mitochondrial membrane potential and induces mitochondrial dysfunction, which is often a key trigger in apoptotic signaling [141,142,143]. For instance, in human leukemia HL60 cells, violacein-induced apoptosis has been associated with specific activation of the TNF receptor 1 pathway [144].

### 5.6. RSSs

RSSs, including H_2_S and Cys-SSH, function as important signaling molecules in eukaryotic systems. In humans, physiological levels of H_2_S can be detoxified to thiosulfate, but excessive H_2_S (due to overgrowth of sulfate-reducing bacteria) can damage epithelial barriers, impair mitochondrial function, and trigger inflammation through elevated cytokine production [145]. Similarly, protozoan parasites such as *E. histolytica* exhibit sensitivity to RSS. Exposure to sodium sulfide (Na_2_S), am H_2_S doner, led to a rapid intracellular uptake of H_2_S and treatment with Cys-SSH causes S-sulfuration of the amebic proteins. This affects post-translational modifications across the parasite proteome and leads to inhibition of protein synthesis, disruption of cytoskeletal organization, reduction in parasitic motility, and inhibition of CPs, which are key virulence factors of *E. histolytica,* thereby compromising the parasite’s virulence [112].

## 6. Conclusions and Future Perspectives

**Figure 1:**
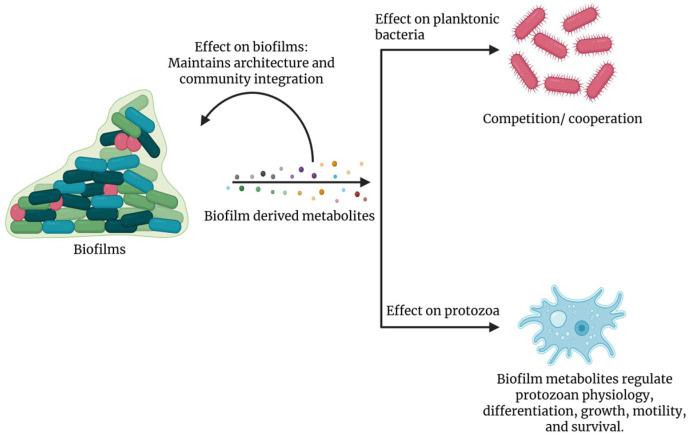
Biofilm-derived compounds mediate multiple ecological interactions. The biofilm community secretes diverse metabolites that support its own structural integrity and maintain community integration. These compounds also influence surrounding planktonic bacteria by promoting cooperative interactions or driving competitive exclusion. In addition, the same metabolites affect protozoan grazers by altering their physiology and reducing their ability to feed on the biofilm.

Biofilm-derived molecules actively coordinate quorum sensing, metabolism, and biofilm organization while providing defense against competing microbes. Additionally, these metabolites also affect protozoan predators by influencing their physiology, modulating motility, encystation, and virulence (Figure 1).

In response, protozoa employ adaptive strategies including metabolic reprogramming, oxidative stress management, and post-translational modifications such as protein S-sulfuration (see Table 1 for summary). Recent studies increasingly highlight the close ecological association between biofilms and protozoa [146,147,148,149], particularly within shared habitats such as the human gut, where parasites such as *E. histolytica* feed on biofilm-associated bacteria. Understanding these interkingdom interactions not only enriches our view of microbial ecology but also offers new opportunities for managing biofilm- and protozoa-related diseases.

Although direct evidence in protozoa remains limited, studies in bacterial and mammalian systems suggest possible mechanisms by which biofilm-derived metabolites could act across kingdoms. For example, in mammalian cells, violacein can induce cytotoxicity via mitochondrial dysfunction, a common upstream trigger of apoptosis. We therefore hypothesize that protozoa may respond to certain biofilm-derived molecules through similar mechanisms. Validating this hypothesis will require ongoing and future research to be focused on elucidating the molecular mechanisms underlying these interactions; specifically, receptors and signaling pathways through which protozoa perceive bacterial metabolites remain an important area of study. Integrating multi-omics approaches with standardized biofilm-protozoa co-culture models will enable detailed exploration of how biofilm metabolites affect protozoan biology and vice versa.

An additional aspect that deserves further attention is the largely anaerobic and reducing nature of the intestinal environment where many bacteria–protozoa interactions occur. Many bacterial metabolites present in the gut are chemically and biologically shaped by low-oxygen conditions, which can strongly influence their stability and mode of action [150,151]. Well-known examples are reactive sulfur species such as hydrogen sulfide that accumulate in reducing environments [152]. In turn, intestinal protozoa rely on metabolic and redox pathways that are highly adapted to anaerobiosis, making them particularly sensitive to changes in redox-active metabolites [153,154]. Future studies should therefore investigate metabolite–protozoa interactions under anaerobic or microaerophilic conditions that better reflect the gut lumen, while also considering oxygen gradients along the mucus–epithelium interface [155]. Importantly, combining anaerobic in vitro culture systems with more physiologically relevant models, such as intestinal organoids [156] or gnotobiotic animals [157], will be essential to capture the complexity of metabolite–protozoa interactions in vivo and to better understand the mechanisms operating in the gut ecosystem.

## Figures and Tables

**Table 1:** Summary of biofilm metabolites involved in interspecies and interkingdom signaling.

Biofilm Compound	Examples	Function in Biofilm	Effect on Bacteria	Effect on Protozoa	References
SCFAs	Acetate, Propionate, Butyrate	Support community structure	Toxic to some; energy source for others	Modulate *Entamoeba* encystation; inhibit *Cryptosporidium*, *Toxoplasma*	[65,66,67,68,69,70]
AHLs	C8-HSL, 3-oxo-C12-HSL, C4-HSL	Regulate biofilm formation, stress, antimicrobials	Mediate cooperation and competition	Affect protozoan behavior; toxic to grazers	[71,122]
Phenazines	PCA, Pyocyanin	Maintain redox balance	Disrupt membranes; induce ROSs	Kill protozoa; stress-induced toxicity	[80,87,88,89]
Indole	-	Interkingdom signaling	Induce stress; limit growth	Disrupt physiology; reduces mitosomal potential	[90,93,135]
Violacein	-	Defense; interspecies signaling	Damage membranes; cause ATP leakage	Cause swelling, lysis; inhibit ATPase	[37,101,139,140]
RSSs	H_2_S, Cys-SSH	Modulate bacterial interactions	Sensitize to antibiotics	Inhibit protein synthesis, motility, and virulence	[111,112,113]

## Data Availability

No new data were created or analyzed in this study. Data sharing is not applicable to this article.

## Abbreviations

The following abbreviations are used in this manuscript:

AHLs	Acyl-homoserine lactones
SCFAs	Short-chain fatty acids
RSS	Reactive sulfur species
ECM	Extracellular matrix
eDNA	Extracellular DNA
AI-2	Autoinducer-2
QS	Quorum sensing
OMVs	Outer membrane vesicles
AIPs	Autoinducing peptides
fMLP	Formyl-methionyl-leucyl-phenylalanine
VOC	Volatile organic compounds
AMPs	Antimicrobial peptides
EPS	Exopolysaccharides
PIA	Polysaccharide intercellular adhesin
ROS	Reactive Oxygen Species
PCA	Phenazine-1-carboxylic acid
TnaA	Tryptophanase
H_2_S	Hydrogen sulfide
Cys-SSH	Cysteine persulfide
